# Mouse movement measures enhance the stop-signal task in adult ADHD assessment

**DOI:** 10.1371/journal.pone.0225437

**Published:** 2019-11-26

**Authors:** Anton Leontyev, Takashi Yamauchi

**Affiliations:** Department of Psychological and Brain Sciences, Texas A&M University,Texas, United States of America; Universita degli Studi di Trento, ITALY

## Abstract

The accurate detection of attention-deficit/hyperactivity disorder (ADHD) symptoms, such as inattentiveness and behavioral disinhibition, is crucial for delivering timely assistance and treatment. ADHD is commonly diagnosed and studied with specialized questionnaires and behavioral tests such as the stop-signal task. However, in cases of late-onset or mild forms of ADHD, behavioral measures often fail to gauge the deficiencies well-highlighted by questionnaires. To improve the sensitivity of behavioral tests, we propose a novel version of the stop-signal task (SST), which integrates mouse cursor tracking. In two studies, we investigated whether introducing mouse movement measures to the stop-signal task improves associations with questionnaire-based measures, as compared to the traditional (keypress-based) version of SST. We also scrutinized the influence of different parameters of stop-signal tasks, such as the method of stop-signal delay setting or definition of response inhibition failure, on these associations. Our results show that a) SSRT has weak association with impulsivity, while mouse movement measures have strong and significant association with impulsivity; b) machine learning models trained on the mouse movement data from “known” participants using nested cross-validation procedure can accurately predict impulsivity ratings of “unknown” participants; c) mouse movement features such as maximum acceleration and maximum velocity are among the most important predictors for impulsivity; d) using preset stop-signal delays prompts behavior that is more indicative of impulsivity.

## Introduction

The capacity for controlling impulsive behavior (i.e., response inhibition) is perhaps the most important function in cognitive control. Clinical, neuropsychological and neuroscientific research suggests that impulsive individuals are likely to be pathological gamblers [1] and engage in drug or alcohol abuse [2].

Impulsivity can be defined and measured in at least two different ways: as a preference for earlier but smaller rewards over later and larger rewards (delay discounting) or as an inability to inhibit undesired actions (behavioral disinhibition) [3]. The difference in definitions provides a challenge for clinicians to diagnose their patients with impulsivity-related disorders such as ADHD (attention-deficit/hyperactivity disorder).

Impulsivity is usually studied using different questionnaires (e.g., Behavior Rating Inventory of Executive Function (BRIEF)). However, questionnaire-based self-reports are prone to various biases, such as cultural [4] or learning [5], as well as an inflated relationship between different facets of executive functioning (i.e., impulsivity and inattention), due to the questionnaires being developed and validated based on one another [6, 7]. Because of these concerns, performance-based methods for studying impulsivity have been applied.

One of the most widely-used performance-based methods is the stop-signal task (SST) [8]. In this task, individuals respond to the stimuli presented in some trials (“go” trials) and withhold a response in trials when a “stop” signal appears (“stop” trials). Poor performance on SST has been related to pathological gambling [9], major depression, anxiety disorder and conduct disorder [10]. Strong associations with performance in this task are found with attention-deficit/hyperactivity disorder (ADHD), obsessive-compulsive disorder (OCD) and schizophrenia [11, 12, 13].

In addition to simple measures such as response time and accuracy, the stop-signal task offers stop-signal reaction time (SSRT), which represents the time that an individual requires to inhibit a “go” response after seeing a “stop” signal. SSRT is known to reflect an individual’s motor inhibition ability [14]; a slower stop-signal reaction time corresponds to damage in the right and left inferior frontal gyrus [15, 16], deep brain stimulation of subthalamic nucleus [17, 18], age-related change in executive functioning [19], cocaine abuse [20] and Parkinson’s disease[21] (for a more comprehensive review, see [22]). SSRT is generally considered to be the best indicator of ADHD among all measures of the stop-signal task [8]. Specifically, children with ADHD were shown to have longer SSRTs [23].

Although SSRT could be applied with healthy individuals to measure the age-related change in inhibitory control [19] or several other experimental manipulations, SSRT as a sole diagnostic tool is limited as it is mostly applicable to individuals with severe clinical disorders. For the general population, the use of this task for clinical application is rather incomplete as SST has minuscule or non-existent correlations with questionnaire-based measures particularly when it is applied to mildly impaired and non-clinical populations [24]. No significant correlations were observed between scores on the Barrat Impulsiveness Scale (BIS-11) and SSRT [25]. Similarly, no significant correlations were found between SSRT and questionnaire-based measures of impulsivity, such as the Eysenck scale, the BRIEF inhibition scale or the ASRS hyperactivity/impulsivity scale [26]. Correlation coefficients between questionnaire-based measures of impulsivity and SSRT or other stop-signal task measures did not exceed .3, with a mean correlation coefficient equal to .05 [27].

This lack of association between questionnaire-based and performance-based measures for healthy individuals is problematic because this prevents researchers from dissecting the functioning of cognitive and affective systems ranging from normal to abnormal, as these measures are not specific and sensitive enough to tease apart the nature of impairment and its variability.

Why are performance-based measures, such as SSRT, so poorly correlated with rating scales? Toplak and colleagues [24] suggest that performance-based tests have poor ecological validity because these tasks are conducted in highly structured environments, and individuals tend to display their best, rather than typical, behavior. In contrast, questionnaires assess typical, everyday behavior. Consequently, performance-based tasks are not indicative of real-life challenges.

The discrepancy between performance-based tests and questionnaires raises a fundamental question. Performance-based tests are essential to probe detailed mechanisms of executive function ranging from normal to abnormal; they offer well-controlled, objective and reproducible measures of behavior. Yet, this highly parameterized design deprives its validity to assess human behavior as it occurs in a natural and typical setting. How can we amend this discrepancy? That is, how can we make a performance-based test, such as SST, more ecologically valid so that the test becomes well aligned to rating scales? That is the topic of this article.

In addition to the aforementioned explanations, we think there is another important reason for the lack of associations between performance- and questionnaire-based measures of impulsivity. Nearly all performance-based tasks reduce the complex working of executive function to simple metrics of response time and accuracy—how fast and accurate one presses a computer key. If impulsivity impacts performance continuously, much information can be lost. We consider reliance on this impoverished data acquisition procedure to be a major culprit for the discrepancy between performance-based and questionnaire-based measures.

This idea is important in light of recent studies pointing to the dynamic nature of the executive functioning, which is reflected in the variability of response times in ADHD participants [28] as well as the dynamic decision conflict resolution that unfolds in two-choice situations [29]. On a neural level, this idea is supported by findings in partial activation of motor neurons during different stages of the decision-making process [30, 31, 32]. Thus, to fully gauge executive functioning in general and behavioral inhibition in particular, performance-based measures that tap into continuous and dynamic characteristics of decision making should be developed.

We think that mouse cursor movement tracking provides a viable remedy. Mouse cursor movement properties, such as the area under the curve, speed and distance have been found to be associated with elevated levels of emotion [33], stress [34], cognitive impairment [35], cognitive load [36, 37], as well as ADHD [7, 38, 39]. Mouse and hand movement properties were also found to be receptive to attention and cognitive control [40, 41] and, most importantly, inhibitory abilities [42].

In the Stroop task, for example, deviation of a cursor trajectory from the straight line between the starting point and the endpoint (e.g., response button) was found to be indicative of congruent or incongruent word color, highlighting the inhibitory processes [42]. In the delay discounting paradigm, mouse movement properties have been related to the degree to which a participant had to overcome an attraction towards an alternative reward [43].

With these observations in mind, the present study has two aims: (1) we devise a mouse tracking version of the stop-signal task and examine the extent to which mouse movement measures improve correlations between SST performance and ADHD impulsivity/inattention ratings, and (2) we identify specific design features that improve SST in association with ADHD rating scales. If mouse movement measures improve SST’s association with ADHD ratings, what trajectory features are most related to ADHD ratings? Which ADHD subscale (e.g., DSM-IV Inattentive symptoms versus DSM-IV: Hyperactive-Impulsive Symptoms) is most correlated with SSRT? In a mouse movement version of SST, how do you define an “incorrect” response in a stop trial in a mouse movement measure? What would be the best way of devising a stop signal delay? Is the standard staircase method superior to a pre-fixed method [44]? The present study addresses these questions.

In two experiments, we contrasted the standard (keypress) and augmented (mouse movement) versions of the stop-signal test and investigated the extent to which mouse movement measures improve associations between SST performance and ADHD rating metrics. In Experiment 1, we applied fixed stop-signal delays [44]. In Experiment 2, we employed a staircase method to implement a stop-signal delay and compared the efficacy of keypress and mouse movement versions of the stop-signal task.

## Experiment 1: Keypress vs. mouse movement (Preset stop-signal delays)

### Methods

All experimental procedures were approved by Texas A&M University Institutional Review Board (IRB, protocol number: IRB2017-0103D). Written consent was obtained from all participants.

#### Participants

A total of 119 Texas A&M undergraduate students who enrolled in an introductory psychology course participated in the experiment for a course credit. They were randomly assigned either to a keypress or a mouse movement condition. Of these participants, 28 did not complete the experiment. Following the procedure employed by Congdon and colleagues [45], we excluded participants who failed to reach at least 5% successful response inhibition in “stop” trials and correctly indicate the coherent dots’ direction in at least 5% of “go” trials. Table 1 summarizes the participants’ demographic information.

**Table 1:** Participants’ demographic characteristics.

		Male	Female	Total
**Keypress**	*N*	25	31	56
	*Age*	19.08 (0.7)	19.25 (1)	19.17 (0.87)
**Mouse motion**	*N*	16	19	35
	*Age*	19.06 (1.12)	19.37 (1.26)	19.22 (1.19)

Values outside and inside parentheses indicate the mean and standard deviations of participants’ age, respectively.

*Procedure*. Participants completed the following tasks in succession:

1. Stop-signal task
2. Conners’ Adult ADHD Rating Scales (CAARS)
3. Barkley Deficits in Executive Functioning Scale (BDEFS for Adults)

#### Stop-signal task

Participants were first required to complete the stop-signal task coupled with a random dot kinematogram [44]. This task consisted of 576 trials, with 432 trials being “go” trials and 144 trials being “stop” trials.

In the stop-signal task, participants were presented with a circular array of 100 white dots moving in the left or right direction. Each dot was 5 pixels in size. Either 10, 50 or 80% of these dots were moving coherently. The remaining dots were moving in random directions. Participants were required to indicate which direction the coherent dots are moving (“go” trials). We chose this task as the “go” task because it allows to study the effect of the “go” stimulus-related information (dot coherency) on behavior in “stop” trials.

On a subset (25%) of trials, a sound signal appeared prompting participants to withhold the movement in this trial (“stop” trials). We chose auditory stop-signal because delivering the stop-signal in this modality has been found to increase the speed and accuracy of stopping processes [46].

Each trial started with a fixation cross that remained on screen for 500 ms. After this, the random dot stimulus appeared for 1100 ms. With the onset of the stimulus, participants had an opportunity to make a response within 3100 ms. In the stop trials, a sound indicating “stop” signal appeared for 100 ms after a delay from the onset of stimulus presentation.

**Fig 1:**
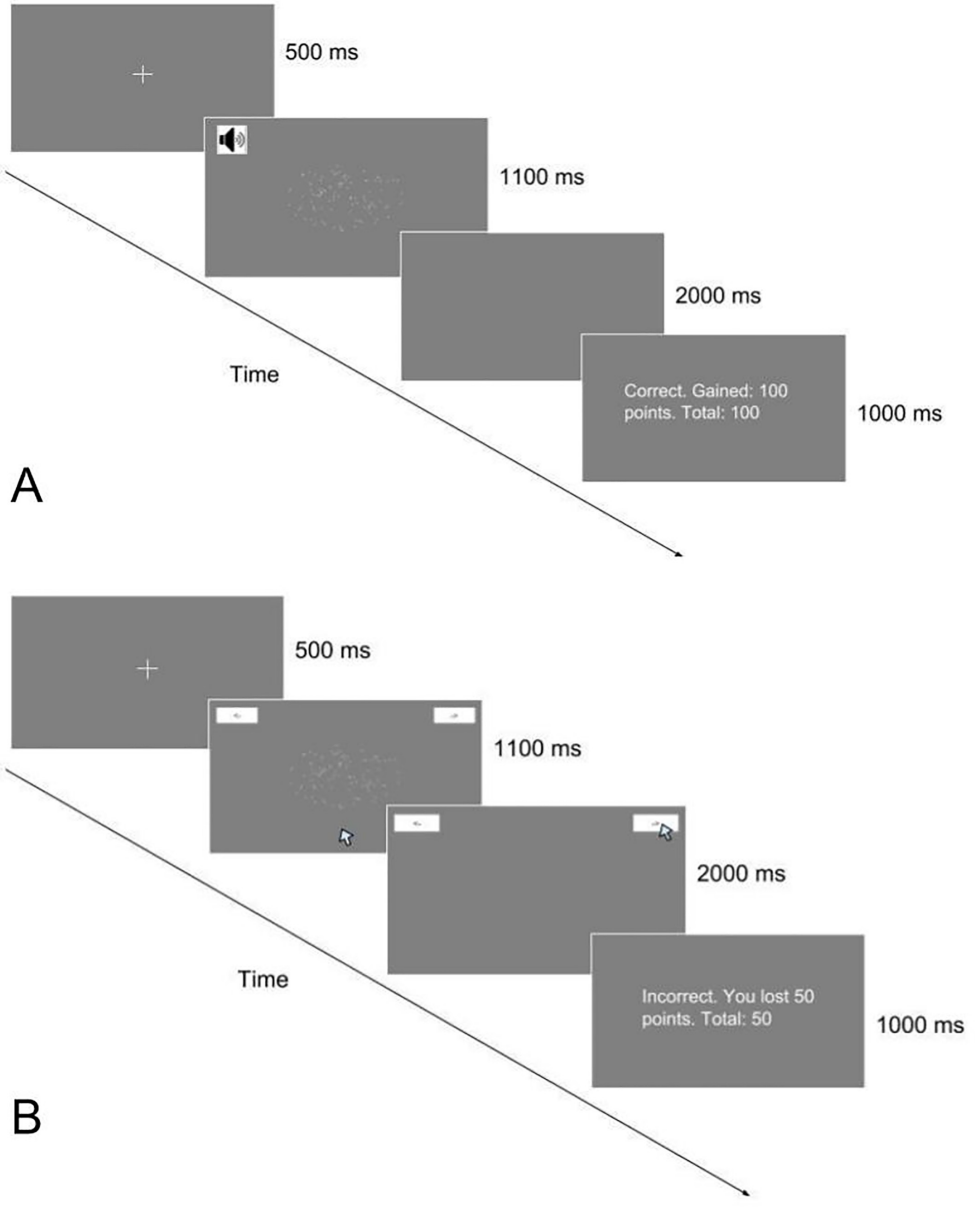
Hypothetical trials in keypress and mouse movement conditions. **A:** Hypothetical trial in the keypress condition. In this trial, a stop-signal (beep) is presented after a 600 ms delay. The participant does not make a response in this example. **B:** Hypothetical trial in the mouse movement condition. In this example, the participant incorrectly identifies the direction of the dot movement as “right”. With the onset of stimulus in each trial in the mouse movement condition, the mouse cursor was placed in the center of the bottom of the screen (0, -0.8), where (0,0) represents the center of the screen in the x-y coordinate system.

Stop-signal delay values were randomly and uniformly chosen from 100, 200, 300, 400, 500 or 600 ms. The trial ended either when participants made a response or 3100 ms since the onset of stimulus have passed. After each trial, participants were given feedback on their performance. Following the procedure employed in the Ma and Yu study [44], participants were given 100 points for a correct response (a correct indication of movement direction or, in stop trials, correct inhibition) and subtracted 50 points for each incorrect response to encourage the best performance. Stimuli and feedback were displayed in white font on a grey background. Fig 1 shows typical trials in both conditions.

The keypress and mouse movement conditions were identical, with an exception for their response collection procedures. In the keypress condition, participants indicated the direction of the dots by pressing a left or right arrow key on the keyboard; in the mouse movement condition participants indicated the direction of the dots by clicking on the button with a left or right arrow drawn. Our program recorded x-y coordinates of the mouse cursor every 16 ms. We used Psychopy [47] software for stimulus presentation and data acquisition.

#### Design

We employed a between-subjects design with one factor (keypress or mouse movement condition). The choice of this design was based on the assumption that a within-subjects design would have increased the number of trials per participant to more than a thousand, potentially inducing fatigue and instructional confusion.

#### Proactive and reactive inhibition measures

There are at least two types of inhibitory control—reactive inhibition and proactive inhibition. Reactive inhibition represents an individual’s ability to inhibit an already initiated motor response, while proactive inhibition is a change in motor strategies “in anticipation of known task demands” (p. 1126) [18]. Proactive inhibition is related to the attenuation of a motor plan relevant to the task [48]. In this study, we assessed both proactive and reactive inhibition, as they are important for ADHD [49].

Research has demonstrated that proactive inhibition can be assessed by the “reaching arm” version of the stop-signal task [50]. Following [21, 44], we measured proactive inhibition with movement initiation time and movement time (the details of these measures are explained later in this section).

To assess reactive inhibition, we measured stop-signal reaction time (SSRT) using the integration method [21]. SSRT is calculated by subtracting SSD (stop signal delay) from the finishing time of the stop process, which is found by integrating the RT distribution and finding the point at which the integral equals the probability of response when a stop-signal is present [50]. Specifically, “go” RTs are rank-ordered and then the n-th “go” RT is selected, with n corresponding to the proportion of inhibitory failures. For example, if the proportion of inhibitory failures (the proportion of making a response in stop trials) is 0.55 for a given participant, n-th “go” RT (finishing time) is the RT equal to the 55th percentile of the “go” RTs. If preset SSDs are used, finishing time is estimated for each SSD and their respective SSDs are then subtracted; to obtain a single SSRT estimate for a given participant, SSRT estimates for different stop-signal delays are averaged [51].

The calculation of SSRT entails defining inhibitory failures (making a response in a “stop” trial). In Experiment 1, we defined “erroneous response” as pressing a key in a stop trial in the keypress condition. In the mouse movement condition, we defined “erroneous response” as participants clicking on a button drawn on the screen in a stop trial.

#### Keypress condition

In the keypress condition, the main independent variables were accuracy, mean and standard deviation of response time and SSRT. Accuracy was calculated for “go” and “stop” trials separately, as well as with respect to participants’ indication of direction (direction discrimination accuracy). Mean and standard deviation of response time were calculated in “go” and “stop” trials separately. In addition, the mean and standard deviation of response times (SD RT) were calculated with respect to three levels of dot coherency (10, 50, or 80% of dots moving in the same direction).

#### Mouse movement condition

In this study, we have aimed to assess stopping impulsivity–a specific form of impulsivity defined [52] as “the tendency to stop an already chosen and initiated, but not fully executed, response” (p.159). Traditionally, this form of impulsivity is assessed through SSRT. However, SSRT is an indirect measure: for example, Verbruggen and Logan [53] consider SSRT an estimate of a covert latency of the stopping process. The continuous design allows for a more direct study of the stopping processes: as Logan and Cowan [54] suggest, “Continuous tasks … offer a way of estimating stop-signal reaction time that does not depend on inhibition functions: The chain of responses stops sometime after the stop signal, and the time between the onset of the stop signal and the occurrence of the last response to be emitted can be used as an estimate of stop-signal reaction time” (p. 319, underscore supplied).

Furthermore, although the horse-race model formalizes the competition between “stop” and “go” processes as a race for a certain threshold, the process of response inhibition is unlikely to be discrete. Shenoy and Yu [55] posit inhibitory control as a dynamic decision-making process, in which an individual “repeatedly assesses the relative value of stopping and going on a fine temporal scale, in order to make an optimal decision on when and whether to go” (p. 1).

Given our aim of studying stopping impulsivity (i.e., stopping an already initiated, but not fully executed, response [45]), findings in dynamic nature of response inhibition and the potential advantages of continuous measurement, we have employed the design different from the one found in the “reaching arm” paradigm [56]. In our version of the stop-signal paradigm, movement can be initiated before the stop-signal is delivered, allowing one to calculate measures that are based on the trajectory of the mouse movement, including mouse movement-based continuous analog of SSRT (i.e., stopping distance, explained later in more detail).

In the mouse movement condition (in addition to the RT and accuracy-based measures) we calculated mean maximum velocity, mean maximum acceleration and mean total distance, separately in go and stop trials and with respect to three levels of dot coherency. Each trajectory was calculated as a sum of distances between adjacent sets of coordinates according to the time of their recording. If 3100 ms passed and the trial ended with no response, all trajectories recorded during the trial were added. No movement was treated as a movement with the distance between coordinates at different timestamps equal to zero. All trajectories were time-normalized using 101 timesteps [57]. For analyzing the cursor trajectory data, we used “mousetrap” package for the R statistical environment [58].

**Fig 2:**
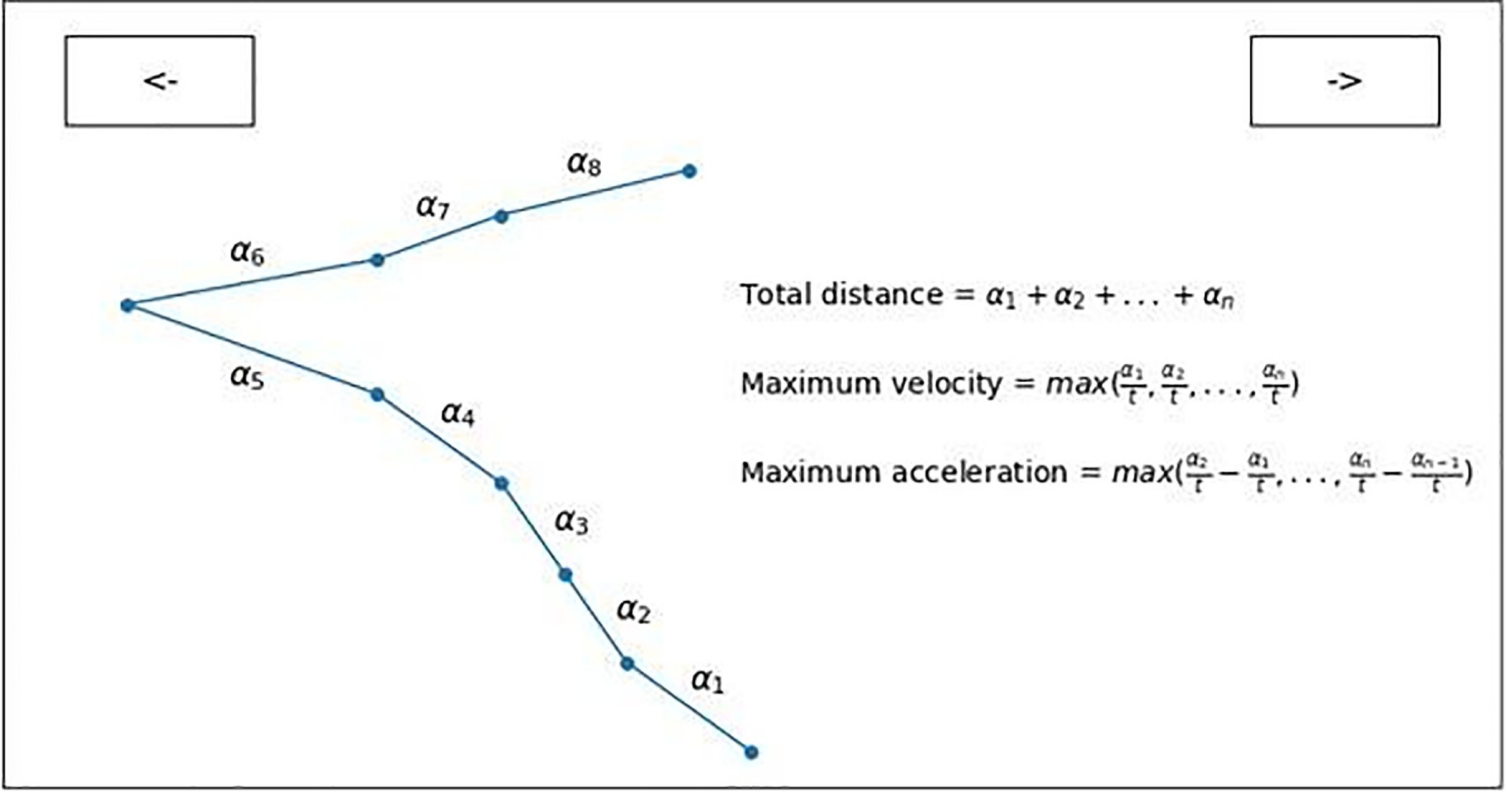
Calculation of trajectory-based measures. Note: *t* represents the time between recordings (≈ 19 ms). Maximum velocity was calculated as the maximum speed with which the movement between two adjacent sets of coordinates took place. Maximum acceleration stands for maximum change in the velocity of the mouse movement between the two adjacent parts of the trajectory. Mean total distance represents the mean of total Euclidean distance from the original starting position of the cursor to the position of the cursor at the end of the trial.

We employed maximum velocity and maximum acceleration (Fig 2) because these measures are likely to reflect levels of commitment, vigor and even impulsiveness for action [59, 60, 61, 7, 62]. Moreover, as the conflict between two options is reflected in the mouse movement [29], mouse movement measures represent the process of resolution of the conflict between “stop” and “go” options.

**Fig 3:**
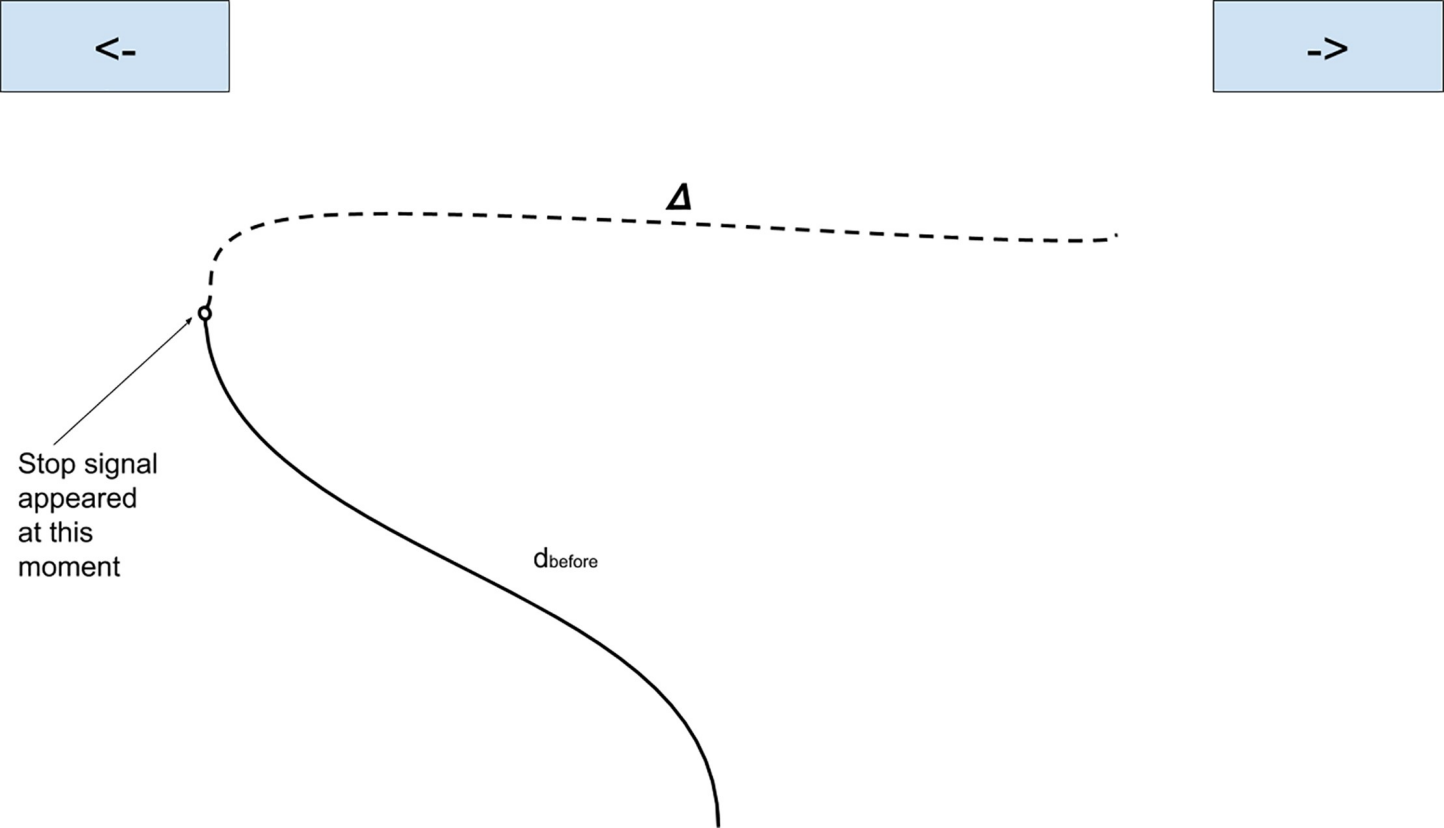
Calculation of stopping distance. *Note*: in this hypothetical trial, an individual continued to move the mouse for some time after hearing the “stop” signal. Stopping distance (*Δ*) is represented by the dashed line. Distance before stop-signal (d_before_) is represented by a solid line.

As a mouse movement measure analog to SSRT, we devised stopping distance (*Δ*). This measure represents the distance that the cursor travels after stop-signal is heard, similar to the time that an individual needs to put the foot on the brake after he or she sees the red traffic light. However, in contrast to SSRT, which is implicit, stopping distance can be directly estimated from a total distance in a trial:

$$\Delta =d_{total}-d_{beforestopsignal},$$

where *d*_*total*_ is total distance an individual has traveled in a given trial, *d*_before stop-signal_ is the distance traveled before stop-signal has appeared and *Δ* is stopping distance (Fig 3).

This measure was calculated separately for each preset stop-signal delay and averaged across all trials for each participant. After that, stopping distances for each stop-signal delay were averaged:

$$\Delta_{average}=\frac{\Delta_{1}+\Delta_{2}+\cdots +\Delta_{n}}{n},$$

where *Δ*_1_,*Δ*_2_…*Δ*_*n*_ are stopping distances calculated for 1^st^, 2^nd^, and nth stop-signal delay, respectively.

When inhibition is not applied, greater acceleration and velocity are achieved [63]. In addition to that, lower maximum velocity and acceleration were linked to responses requiring more top-down control, e.g. ones that required the deception [64]. Stopping distance cannot be attributed to the muscular constraints but is rather a result of higher-level planning [65].

For studying proactive inhibition, we employed, following Mirabella and colleagues [21], movement initiation time and movement time. Movement initiation time was calculated relative to the onset of the random dot kinematogram. Movement time was calculated as the difference between movement initiation time and time when a participant clicked the response button or the trial ended and the recording stopped. These measures were calculated only for the “go” trials since proactive inhibition can manifest as slowing the response in anticipation of the “stop” signal [66].

Results. To make sure samples collected in keypress and mouse movement conditions are comparable in their ADHD and impulsivity/inattention levels, we first analyzed the differences in distributions of scores on different questionnaire scales between two samples. We also investigated the correlations between impulsivity/inattention scores and performance in the stop-signal task, separately in keypress and mouse movement conditions. Finally, we applied ridge regression to assess the predictive ability of the keypress and mouse movement measures and find the features that are most strongly related to inclination towards different subsets of ADHD.

#### Distributions of CAARS & BDEFS scores in keypress and mouse movement conditions

Two-sample Kolmogorov-Smirnov test showed no difference in distributions of questionnaire scores between keypress and mouse movement conditions on any subscale.

#### Correlation analysis

To investigate the correlations between performance-based measures and impulsivity/inattention profile, we used Spearman’s rank correlation. Following the Ma and Yu study [44], we also examined the extent to which different non-inhibition-related factors affect the associations between performance measures and questionnaire-based measures of impulsivity and inattention. Specifically, we examined the changes in associations between SSRT and other measures and scores on different subscales across different levels of dot coherency.

#### Correlations between performance and impulsivity measures in keypress condition

In the keypress condition, we have found a few correlations between RT- or accuracy-based measures and scores on the questionnaires. Specifically, significant correlations were observed between mean RT in “stop” trials and scores indicating an inclination towards Inattentive subtype of ADHD (*ρ* = -0.27, *p* = .04), as well as between accuracy in “stop” trials and Impulsivity/Emotional lability subscale (*ρ* = -0.29, *p* = .03). Correlations with impulsivity and inattention measures are summarized in Table 2.

**Table 2:** Values of Spearman’s ρ for correlations between keypress measures and impulsivity/inattention measures.

	Impulsivity/Emotional lability	DSM-IV: Hyperactive/Impulsive symptoms	Inattention/Memory problems	DSM-IV: Inattentive
**RT in “go”**	-0.09	-0.07	-0.15	-0.22
**SD RT in “go”**	0.18	0.08	0.02	-0.03
**Accuracy in “go”**	0.14	0.22	0.19	0.08
**RT in “stop”**	-0.06	-0.04	-0.2	-0.27*
**SD RT in “stop”**	0.09	0.03	-0.1	-0.09
**Accuracy in “stop”**	-0.29*	0.01	-0.14	-0.13
**Direction discrimination accuracy**	-0.06	0.13	-0.02	-0.15
**SSRT**	0.1	-0.05	-0.08	-0.2

* p < .05, ** p < .01, *** p <. 001.Direction discrimination accuracy refers to the proportion of “go” trials in which a participant correctly indicated the dot movement direction.

After separating trials by different dot coherency levels (10%, 50% and 80%), significant correlations were found between stop-signal reaction time at 10% coherency and inclination towards Combined subtype of ADHD (*ρ* = -0.34, *p* = .01), as well as ADHD index (*ρ* = -0.28, *p* = .04). Among BDEFS subscales, SSRT at 10% coherence was negatively correlated with Self-Management to Time (*ρ* = -0.27, *p* = .043). Correlations between RT-based measures and scores on impulsivity/inattention scales are summarized in Table 3.

**Table 3:** Values of Spearman’s ρ for correlations between keypress measures and impulsivity/inattention measures at different coherence levels.

	Impulsivity/Emotional lability	DSM-IV: Hyperactive/Impulsive symptoms	Inattention/Memory problems	DSM-IV: Inattentive
**RT in “go” at 10%**	-0.09	-0.07	-0.15	-0.22
**RT in “stop” at 10%**	-0.06	-0.01	-0.12	-0.17
**SSRT at 10%**	0.006	-0.05	-0.18	-0.21
**RT in “go” at 50%%**	-0.08	-0.07	-0.16	-0.22
**RT in “stop” at 50%**	-0.14	-0.09	-0.28*	-0.28*
**SSRT at 50%**	0.08	-0.03	-0.14	-0.21
**RT in “go” at 80%**	-0.09	-0.07	-0.14	-0.21
**RT in “stop” at 80%**	0.02	0.008	-0.16	-0.26
**SSRT at 80%**	0.18	0.04	0.01	-0.03

* p < .05, ** p < .01, *** p < .001. The scores in italics represent statistically significant correlations after controlling the false discovery rate at q = 0.05 [67].

#### Correlations between performance and impulsivity measures in the mouse movement condition

In contrast to the keypress condition, we have found multiple correlations between mouse movement measures and impulsivity/inattention scores.

Simple RT- and accuracy-based measures collected in mouse movement condition had no significant correlations with impulsivity measures, but had a few significant correlations with inattention measures, summarized in Table 4:

**Table 4:** Values of Spearman’s ρ for correlations between simple RT/ accuracy-based measures and impulsivity/inattention measures in mouse movement condition.

	Impulsivity/Emotional lability	DSM-IV: Hyperactive/Impulsive symptoms	Inattention/Memory problems	DSM-IV: Inattentive
**RT in “go”**	-0.06	0.13	0.08	0.08
**SD RT in “go”**	0.01	0.07	0.05	0.19
**Accuracy in “go”**	0.15	-0.04	-0.13	-0.12
**RT in “stop”**	0.04	-0.04	0.11	0.11
**SD RT in “stop”**	-0.24	-0.14	0.03	0.16
**Accuracy in “stop”**	-0.07	-0.14	-0.4*	-0.35*
**Direction discrimination accuracy**	-0.02	-0.28	-0.08	-0.1
**SSRT**	0.18	0.28	0.41*	0.34*

* p < .05, ** p < .01, *** p < .001. Direction discrimination accuracy refers to the proportion of “go” trials in which a participant correctly indicated the dot movement direction.

Among mouse movement-specific measures, significant correlations were observed between BDEFS Section 5—Self-regulation of emotion and accuracy in “stop” trials (*ρ* = -0.38, *p* = .025) and BDEFS Section 5 and SSRT (*ρ* = 0.35, *p* = .04) Among mouse-specific measures, the strongest associations were found between scores on subscale C–Impulsivity/Emotional lability–and average stopping distance (*ρ* = 0.43, *p* = .01). Scores on this subscale were also significantly correlated with mean total distance in “go” (*ρ* = 0.34, p = .044) and “stop” (*ρ* = 0.38, p = .024) trials, as well as mean maximum acceleration in “stop” trials (*ρ* = 0.36, *p* = .034). Inclination towards a primarily impulsive subtype of ADHD (subscale F) was significantly correlated with mean maximum velocity in “go” (*ρ* = -0.45, *p* = .006) and “stop” (*ρ* = -0.44, *p* = .009) trials. Table 5 summarizes the correlations between mouse movement measures and impulsivity measures.

**Table 5:** Values of Spearman’s ρ for correlations between mouse movement and impulsivity/inattention measures.

	Impulsivity/Emotional lability	DSM-IV: Hyperactive/Impulsive symptoms	Inattention/Memory problems	DSM-IV: Inattentive
**Velocity in “go”**	0.2	*-0*.45**	0.09	-0.05
**Acceleration in “go”**	0.29	-0.08	-0.2	-0.17
**Total distance in “go”**	0.34**	-0.19	0.03	0.06
**Velocity in “stop”**	0.03	*-0*.44*	0.14	-0.02
**Acceleration in “stop”**	0.36*	0.03	-0.16	-0.02
**Total distance in “stop”**	0.38*	0.03	-0.16	-0.02
**Stopping distance**	*0*.43**	0.18	-0.18	0.04
**Initiation time in “go”**	-0.15	0.01	0.01	-0.03
**Movement time**	-0.03	0.16	0.12	0.16

* p < .05

** p < .01, *** p < .001. The scores in italics represent statistically significant correlations after controlling the false discovery rate at q = 0.05 [67].

After separating trials by different dot coherency levels (10%, 50%, and 80%), significant correlations appeared between scores subscale C and total distance in “go” and “stop” trials at 10% and 50% levels of dot coherency. Impulsivity/Emotional lability was also significantly correlated with mean maximum acceleration in stop trials at 50% (*ρ* = 0.47, *p* = .004). In contrast with keypress condition, a measure analogous to SSRT–average stopping distance–was significantly correlated with Impulsivity/Emotional lability scores at all levels of coherency.

Inclination towards Impulsive subtype (subscale F) showed significant correlations with mean maximum velocity in “go” and “stop” trials at all levels of coherency. Tables 6–8 summarize correlations between mouse movement measures and scores on subscales C (Impulsivity/Emotional lability), F (DSM-IV: Hyperactive/Impulsive symptoms), A (Inattention/Memory problems) and E (DSM-IV: Inattentive symptoms) across different levels of dot coherency.

**Table 6:** Values of Spearman’s ρ for correlations between mouse movement and impulsivity/inattention measures at 10% coherency level.

	Impulsivity/Emotional lability	DSM-IV: Hyperactive/Impulsive symptoms	Inattention/Memory problems	DSM-IV: Inattentive
**Velocity in “go”**	0.03	*-0*.46**	0.08	-0.03
**Acceleration in “go”**	0.27	-0.0	-0.22	-0.17
**Total distance in “go”**	0.36*	-0.18	-0.05	0.07
**Velocity in “stop”**	0.08	-0.35*	0.14	-0.03
**Acceleration in “stop”**	0.31	-0.01	-0.16	-0.11
**Total distance in “stop”**	0.35*	0.1	-0.08	0.11
**Stopping distance**	*0*.42*	0.17	-0.18	0.05

* p < .05

** p < .01, *** p < .001. The scores in italics represent statistically significant correlations after controlling the false discovery rate at q = 0.05 [67].

**Table 7:** Values of Spearman’s ρ for correlations between mouse movement and impulsivity/inattention measures at 50% coherency level.

	Impulsivity/Emotional lability	DSM-IV: Hyperactive/Impulsive symptoms	Inattention/Memory problems	DSM-IV: Inattentive
**Velocity in “go”**	0.02	*-0*.44**	0.12	-0.06
**Acceleration in “go”**	0.24	-0.13	-0.22	-0.21
**Total distance in “go”**	0.34*	-0.18	-0.22	-0.21
**Velocity in “stop”**	-0.02	*-0*.45**	0.15	-0.01
**Acceleration in “stop”**	*0*.47**	0.12	-0.14	-0.13
**Total distance in “stop”**	*0*.46**	0.08	-0.12	-0.01
**Stopping distance**	*0*.45**	0.15	-0.17	0.02

* p < .05

** p < .01, *** p < .001. The scores in italics represent statistically significant correlations after controlling the false discovery rate at q = 0.05 [67].

**Table 8:** Values of Spearman’s ρ for correlations between mouse movement and impulsivity/inattention measures at 80% coherency level.

	Impulsivity/Emotional lability	DSM-IV: Hyperactive/Impulsive symptoms	Inattention/Memory problems	DSM-IV: Inattentive
**Velocity in “go”**	0.08	*-0*.41*	0.11	-0.01
**Acceleration in “go”**	0.29	-0.08	-0.18	-0.23
**Total distance in “go”**	0.3	-0.19	0.03	0.07
**Velocity in “stop”**	0.1	*-0*.41*	0.1	-0.02
**Acceleration in “stop”**	0.31	-0.03	-0.19	-0.13
**Total distance in “stop”**	0.33	0.01	-0.24	-0.8
**Stopping distance**	*0*.43**	0.17	-0.18	0

* p < .05

** p < .01, *** p < .001. The scores in italics represent statistically significant correlations after controlling the false discovery rate at q = 0.05 [67].

#### Feature selection and prediction performance

So far, our analysis focused on correlations between performance measures (e.g., maximum velocity in stop trials) and ADHD ratings (hyperactive/impulsive symptoms). These correlation measures show the degree of association between two sets of data. It is unclear how well one can predict hypothetical questionnaire scores based on the performance measures (collected in keypress or mouse movement conditions). It is also unclear which performance features are related to different ADHD subtypes. Mouse movement features, such as maximum velocity and maximum acceleration, are by design correlated. This intercorrelation poses a risk of multicollinearity. To investigate these questions, we applied ridge regression and conducted feature subset selection and model assessment following the procedure delineated by James and colleagues [68].

#### Feature subset selection

For the feature subset selection, we applied ridge regression. Ridge regression is a regression technique especially useful when data suffer from multicollinearity [69]. Multicollinearity occurs when some predictors are strongly correlated with others. When it happens, the variance of coefficient estimates can dramatically increase.

Ridge regression reduces the coefficient estimates using a regularization parameter, *λ*. To choose the best *λ*, we employed 10-fold cross-validation [68]. Each data set was divided into 10 segments (folds 1–10). One segment was left for the test (fold 1), and the remaining segments (folds 2–10) were used for training. In the training segments (folds 2–10), models with various lambda values are assessed and tested with the test data (fold 1). This process was repeated 10 times with other folds, and the lambda that yielded the minimum mean squared error with the test data was selected. In both keypress and mouse movement conditions, we standardized the predictors (mean = 0, SD = 1).

**Fig 4:**
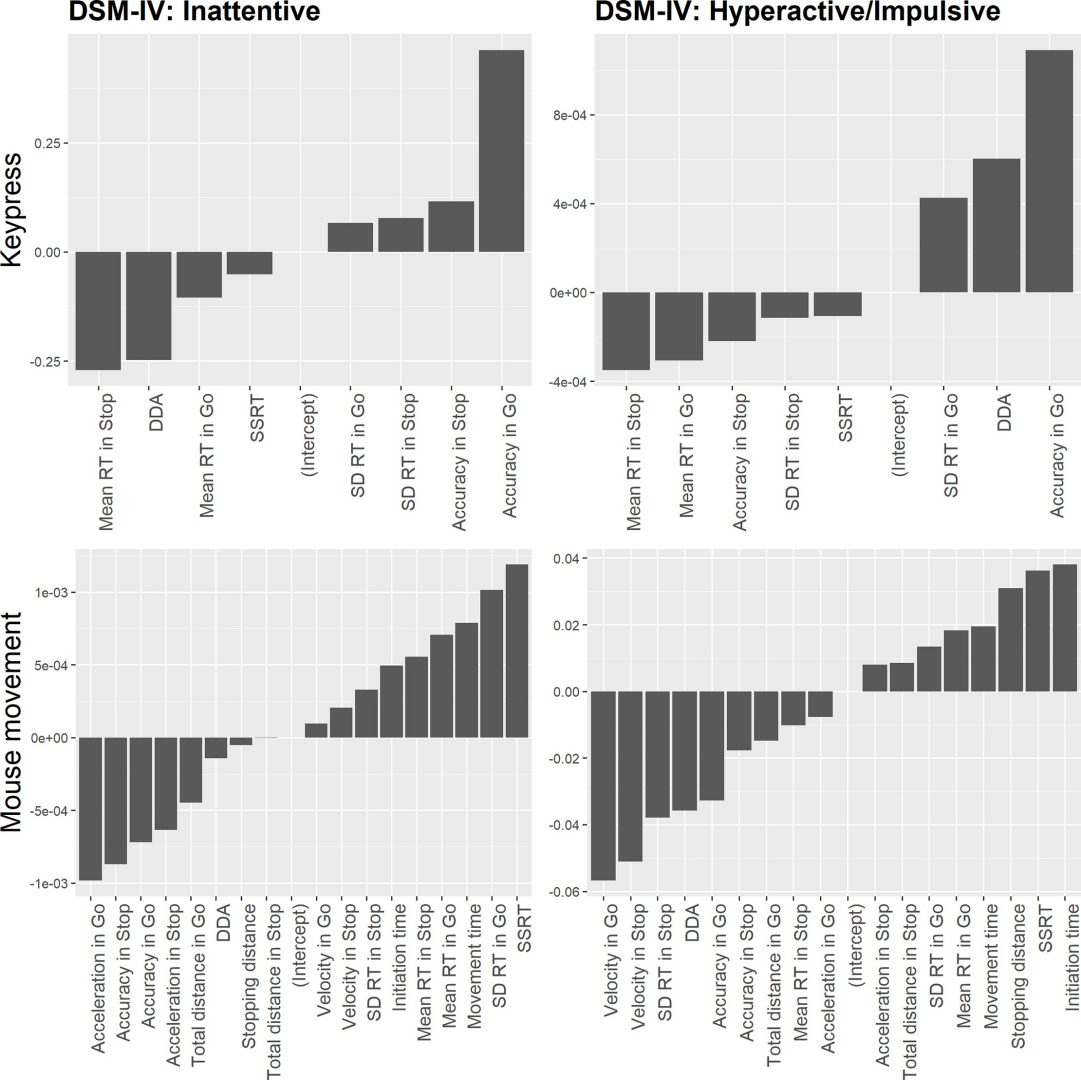
Ridge regression coefficients in keypress and mouse movement conditions. *Note*: coefficient value closer to zero indicates less importance. DDA = Direction discrimination accuracy.

In the keypress condition, the most important features for subscale F (DSM-IV: Hyperactive/Impulsive symptoms) were accuracy in “go” trials and SSRT. Accuracy in “go” trials and direction discrimination accuracy were also indicative of subscale E (DSM-IV: Inattentive symptoms) (Fig 4). In the mouse movement condition, the most important features for subscale F (DSM-IV: Hyperactive/Impulsive symptoms) were mean maximum velocity in “go” trials and stopping distance. For subscale E (DSM-IV: Inattentive symptoms), the most important features were acceleration in “go” trials and SSRT.

A comparison of coefficients’ absolute values between keypress and mouse movement conditions reveals that for Hyperactive/Impulsive symptoms subscale, features in mouse movement condition had larger absolute values. In contrast, features in keypress condition had greater coefficients’ value for Inattentive symptoms subscale.

#### Evaluation of prediction performance

What is the prediction capacity of these features? That is, given “new” subjects, to what extent can these measures predict ADHD ratings of the “unknown” subjects? In order to evaluate the prediction performance of models in keypress and mouse movement, we applied the nested cross-validation (CV) method—10-fold cross-validation nested within 5-fold cross-validation (5-fold CV), combined with bootstrapping sampling. In the outer layer of cross-validation (5-fold CV), fold 1 was left for the test, while folds 2–5 were used for model selection. Within the training data (folds 2–5), we applied the 10-fold CV to find the best lambda as described in the feature selection section. The model (λ and coefficients) thus identified in the 10-fold CV the training data (folds 2–5 in the 5-fold CV) were assessed by the test data (fold 1 in the 5-fold CV). This process was cycled over other folds (e.g., fold 2 for test and folds 1, 3, 4, 5 for training, and so on) and repeated 1000 times with bootstrapped samples (in each iteration of 5-fold CV, samples were selected randomly from original data with replacement). In this manner, we separated the model selection and model assessment completely and examined the extent to which identified models can infer ADHD ratings of “new” pseudo-participants.

As Table 9 shows, the models formed in the mouse movement condition performed well for the subscales C (Impulsivity/Emotional lability) and F (DSM-IV: Hyperactive/Impulsive symptoms). Given subscale E (DSM-IV: Inattentive symptoms), the model formed in the keypress condition showed good prediction performance.

**Table 9:** Spearman’s ρ for correlations between predicted and test data in keypress and mouse movement conditions for scores on impulsivity and inattentiveness subscales.

	Impulsivity/Emotional lability	DSM-IV: Hyperactive/Impulsive symptoms	Inattention/Memory problems	DSM-IV: Inattentive symptoms
Keypress	0.27 (0.23)	0.03 (0.24)	0.24 (0.26)	0.29 (0.21)
Mouse movement	0.29 (0.28)	0.24 (0.34)	0.12(0.36)	-0.008 (0.33)

Values outside the parentheses illustrate the median values of *ρ*. Values inside the parentheses illustrate the median absolute deviations of *ρ*.

#### Discussion

In Experiment 1, we found that measures collected in mouse movement-based stop-signal task had more significant correlations with questionnaire measures of impulsivity and inattention than those collected in the traditional keypress-based stop-signal task.

In the keypress condition, no significant correlations were observed with scores on subscales C (Impulsivity/Emotional lability) or F (DSM-IV: Impulsive subtype). In addition to that, as the results of ridge regression analysis show, keypress measures better predicted (by the measure of the correlation between predicted and test data) scores on subscales A (Inattention/Memory problems) and E (DSM-IV: Inattentive symptoms). Moreover, as feature selection shows, coefficients with the largest absolute value were produced for Inattentive symptoms subscale.

In contrast, mouse movement measures, particularly mean maximum velocity and stopping distance, were correlated with CAARS scores on the inclination towards a primarily Impulsive subtype. For Inattentive symptoms, as well as for inclination towards the Inattentive subtype of ADHD, no mouse movement measures had any significant correlation with questionnaire scores. Furthermore, in contrast with the models in the keypress condition, models in mouse movement condition were good at predicting scores on impulsivity-related subscales: C (Impulsivity/Emotional lability) and F (DSM-IV: Hyperactive/Impulsive symptoms). Coefficients with the absolute value farthest away from zero for models in mouse movement condition were produced for Hyperactive/Impulsive symptoms subscale.

In the traditional horse-race inhibition model [53], “go” and “stop” processes are assumed to be independent (i.e., processes affecting “go” processes do not affect “stop” processes) (but see Ma & Yu [44], Logan et al. [70] and Schall, Palmieri and Logan [71]). Thus, dot coherency was supposed to be relevant only for “go” trials to correctly indicate the direction of the coherent dot movement. However, in this experiment, dot coherency in “stop” trials influenced correlations with CAARS scores. We found that in both the keypress and mouse movement conditions, correlations between performance in “stop” trials and questionnaire-based measures depended on dot coherency. For example, velocity and acceleration in “stop” trials had significant correlations with questionnaire-based measures of impulsivity on the 50% dot coherency level, but not 10 or 80%.

Given that, the influence of dot coherence on behavior in “stop” trials is vexing. At this point, we do not have a clear explanation for why dot coherency impacted the association between stop-signal task performance and ADHD questionnaire scores. It is possible that, in order for the stop-signal task to be effective, the secondary task (i.e., judging the left-right direction of random dots) needs to be reasonably engaging. Thus, judgment processes for go trials and stop trials are mutually dependent.

Following the finding that control processes involving go and stop judgment are continuous and inseparable [44], Experiment 1 employed the integration method to calculate SSRT. The advantage of the mouse movement measures as an impulsivity-related assessment tool may be limited to this special circumstance. Although previous studies reported no difference between SSRTs calculated with fixed-SSD and staircase methods of setting SSDs [72], the difference can be attributed to the different response strategies employed by participants. Specifically, in the staircase method condition, participants might tend to choose to wait for the stop-signal more, allocating more resources to the motor inhibition [73]. If an individual chooses this strategy, it is likely that both response time and mouse movement measures would be affected as well.

In Experiment 2, we employed the staircase method of calculating stop-signal delays and contrasted the keypress and mouse movement measures in impulsivity assessment.

## Experiment 2: Keypress vs. mouse movement (stop-signal delays set using the staircase method)

The design and procedure of Experiment 2 were identical to those described in Experiment 1, except that we employed a staircase method to implement stop-signal delays and a different way of defining inhibition failure.

### Methods

#### Participants

A total of 113 Texas A&M undergraduate students who enrolled in an introductory psychology course participated in the experiment for course credit. They were randomly assigned either to a keypress or mouse movement condition. Of these participants, 13 did not complete the experiment. As in Experiment 1, we excluded participants who failed to reach at least 5% successful response inhibition in “stop” trials and correctly indicate the coherent dots’ direction in at least 5% of “go” trials. Table 10 summarizes the participants’ demographic information.

**Table 10:** Participants’ demographic characteristics.

		Male	Female	Total
***Keypress***	*N*	27	23	50
	*Age*	18.7 (0.9)	18.5 (0.98)	18.64(1.04)
***Mouse motion***	*N*	15	35	50
	*Age*	19.1 (1.93)	18.8 (1.11)	18.96 (1.6)

Values outside and inside parentheses indicate the mean and standard deviations of participants’ age, respectively.

#### Design

**Fig 5:**
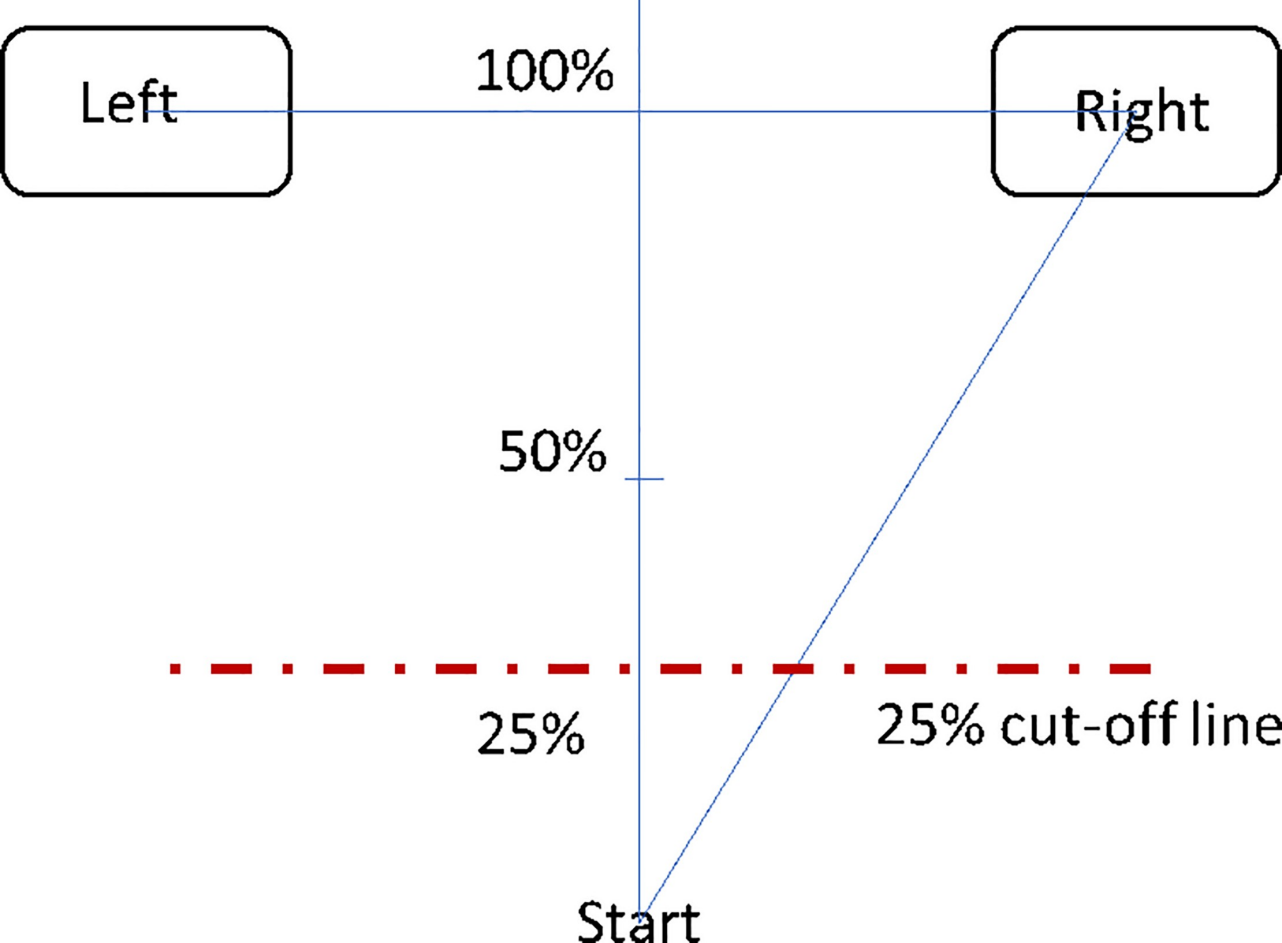
Response inhibition criteria. Red dashed line drawn at 25% of the vertical distance connecting the start position and an end button depicts the cutoff for the response in a “stop” trial.

The design of Experiment 2 was identical to that of Experiment 1 with a few exceptions: In Experiment 2, we employed a staircase method to calculate a stop-signal delay. When a participant made a successful inhibition (e.g., a participant not responding in a “stop” trial), stop-signal delay (SSD) was increased by 50 ms. Conversely, when the participant failed to inhibit the response in a “stop” trial, SSD was decreased by 50 ms [74]. In the mouse movement condition, a failure of inhibition (i.e., making a response in a stop trial) was defined as the participant moving the cursor beyond a cut-off line of a 25% of the vertical distance connecting the centers of the start button and an end button (Fig 5). The initial SSD was set at 600 ms for all participants, which corresponds to “stop” accuracy of 50% in Experiment 1. To calculate SSRT, we calculated overall finishing time by rank-ordering RTs in “go” trials and selecting RT equal to nth percentile, where n is equal to the proportion of inhibitory failures. We then averaged SSDs over all stop-signal trials and subtracted the mean SSD from the finishing time to provide a subject-level SSRT estimate [51].

Because BDEFS questionnaires were not correlated with any of the response time, accuracy, and mouse movement measures, BDEFS measures were not included in Experiment 2.

#### Results

We first investigated the differences in distributions of scores on different questionnaire scales between the two samples. Then, we analyzed correlations and predictive abilities of regression models describing the relationship between impulsivity/inattention scores and performance measures and identified the most important features. These analyses were performed separately in keypress and mouse movement conditions.

#### Distributions of CAARS scores in keypress and mouse movement conditions

Save for subscales C (Impulsivity/Emotional lability) (*d* = 0.28, *p* = .04) and E (DSM-IV: Inattentive symptoms) (*d* = 0.28, *p* = .04), no significant differences in scores between two conditions were observed.

#### Correlation analysis

Similar to the previous experiment, we employed Spearman’s rank correlation due to its robustness to outliers. We also included different dot coherency levels in our analysis.

#### Correlations between performance and impulsivity measures in keypress condition

In the keypress condition, most significant correlations with questionnaire scores were found only with accuracy in “go” trials (Table 11).

**Table 11:** Values of Spearman’s ρ for correlations between keypress measures and impulsivity/inattention measures.

	Impulsivity/Emotional lability	DSM-IV: Hyperactive/Impulsive symptoms	Inattention/Memory problems	DSM-IV: Inattentive
**RT in “go”**	0.22	0.01	0.17	0.17
**SD RT in “go”**	-0.05	0.13	0	-0.09
**Accuracy in “go”**	*-0*.43**	-0.27	-0.22	-0.07
**RT in “stop”**	0.2	0.03	0.14	0.19
**SD RT in “stop”**	-0.03	0.21	-0.01	0.06
**Accuracy in “stop”**	0.15	0.01	0.05	0.04
**Direction discrimination accuracy**	*-0*.44**	-0.26	-0.33*	-0.15
**SSRT**	-0.22	0.04	-0.03	-0.13

* p < .05

** p < .01, *** p < .001. The scores in italics represent statistically significant correlations after controlling the false discovery rate at q = 0.05[67]. Direction discrimination accuracy refers to the proportion of “go” trials in which a participant correctly indicated the dot movement direction.

After separating trials by dot coherency levels, no significant correlations between mean RT or SSRT and questionnaire scores were observed (Table 12).

**Table 12:** Values of Spearman’s ρ for correlations between keypress measures and impulsivity/inattention measures at different coherence levels.

	Impulsivity/Emotional lability	DSM-IV: Hyperactive/Impulsive symptoms	Inattention/Memory problems	DSM-IV: Inattentive
**RT in “go” at 10%**	0.21	0.02	0.14	0.17
**RT in “stop” at 10%**	0.24	0.01	0.12	0.14
**SSRT at 10%**	-0.08	0.07	-0.13	0.13
**RT in “go” at 50%**	0.17	-0.03	0.13	0.12
**RT in “stop” at 50%**	0.15	0.04	0.16	0.2
**SSRT at 50%**	-0.12	-0.17	-0.03	0
**RT in “go” at 80%**	0.19	0.02	0.15	0.18
**RT in “stop” at 80%**	0.22	0.04	0.16	0.19
**SSRT at 80%**	-0.08	0.08	-0.1	-0.03

* p < .05, ** p < .01, *** p < .001.

***Correlations between performance and impulsivity measures in mouse movement condition*.** In contrast, we found a number of significant correlations in the mouse movement condition. Among simple RT- and accuracy-based measures, correlations appeared mostly with inattention measures. These correlations are summarized in Table 13.

**Table 13:** Values of Spearman’s ρ for correlations between simple RT/ accuracy-based measures and impulsivity/inattention measures in mouse movement condition.

	Impulsivity/Emotional lability	DSM-IV: Hyperactive/Impulsive symptoms	Inattention/Memory problems	DSM-IV: Inattentive
**RT in “go”**	-0.16	-0.32*	-0.28*	-0.31*
**SD RT in “go”**	-0.08	0.1	-0.37**	-0.2
**Accuracy in “go”**	0.13	0.11	0.16	0.06
**RT in “stop”**	-0.12	-0.27	-0.27	-0.27
**SD RT in “stop”**	0.02	-0.14	-0.13	-0.05
**Accuracy in “stop”**	-0.21	-0.29*	-0.22	-0.28*
**Direction discrimination accuracy**	0.11	-0.01	0.13	-0.05
**SSRT**	0.1	0.08	-0.02	-0.12

* p < .05

** p < .01, *** p < .001. Direction discrimination accuracy refers to the proportion of “go” trials in which a participant correctly indicated the dot movement direction.

After calculating response time measures by coherency levels, however, significant correlations appeared between response time in “go” trials and impulsivity/inattention measures (Table 14):

**Table 14:** Values of Spearman’s ρ for correlations between simple RT/ accuracy-based measures and impulsivity/inattention measures in the mouse movement condition calculated at different coherency levels.

	Impulsivity/Emotional lability	DSM-IV: Hyperactive/Impulsive symptoms	Inattention/Memory problems	DSM-IV: Inattentive
**RT in “go” at 10%**	-0.16	-0.32*	-0.28	-0.33*
**RT in “stop” at 10%**	-0.12	-0.26	-0.28	-0.27
**SSRT at 10%**	0.05	0.04	-0.08	-0.16
**RT in “go” at 50%**	-0.17	-0.32*	-0.3*	-0.31*
**RT in “stop” at 50%**	-0.13	-0.27	-0.26	-0.26
**SSRT at 50%**	0.07	0.03	-0.05	-0.19
**RT in “go” at 80%**	-0.14	-0.29*	-0.26	-0.29*
**RT in “stop” at 80%**	-0.07	-0.23	-0.24	-0.25
**SSRT at 80%**	0.27	0.19	0.08	0.05

* p < .05, ** p < .01, *** p < .001.

Among mouse movement-specific measures, such as velocity, acceleration, and total distance, most correlations appeared with inattention measures, albeit a few strong ones were also found with impulsivity measures (Table 15).

**Table 15:** Values of Spearman’s ρ for correlations between mouse movement and impulsivity/inattention measures.

	Impulsivity/Emotional lability	DSM-IV: Hyperactive/Impulsive symptoms	Inattention/Memory problems	DSM-IV: Inattentive
**Velocity in “go”**	0.15	0.13	0.11	0.18
**Acceleration in “go”**	0.1	0.1	0.06	0.15
**Total distance in “go”**	0.26	0.16	0.18	0.23
**Velocity in “stop”**	0.29*	*0*.35*	0.24	*0*.43**
**Acceleration in “stop”**	0.27	0.32*	0.17	*0*.40**
**Total distance in “stop”**	0.08	0.13	0.11	0.26
**Initiation time in “go”**	-0.04	-0.13	-0.12	-0.11
**Movement time**	-0.25	-0.24	-0.27	-0.26

* p < .05

** p < .01, *** p < .001. The scores in italics represent statistically significant correlations after controlling the false discovery rate at q = 0.05[67].

After calculating these measures separately on different levels, significant correlations were found among measures calculated based on trials with 50% coherency, but not 10 or 80% coherency (summarized in Tables 16–18).

**Table 16:** Values of Spearman’s ρ for correlations between mouse measures and impulsivity/inattention measures at 10% coherence.

	Impulsivity/Emotional lability	DSM-IV: Hyperactive/Impulsive symptoms	Inattention/Memory problems	DSM-IV: Inattentive
**Velocity in “go”**	-0.01	0.1	-0.02	0.13
**Acceleration in “go”**	0.03	0.18	-0.05	0.12
**Total distance in “go”**	-0.04	0.05	-0.08	0.06
**Velocity in “stop”**	0.15	0.28	0.07	0.23
**Acceleration in “stop”**	0.06	0.23	0.04	0.26
**Total distance in “stop”**	-0.07	0.14	-0.06	0.15

* p < .05, ** p < .01, *** p < .001.

**Table 17:** Values of Spearman’s ρ correlations between mouse measures and impulsivity/inattention measures at 50% coherency level.

	Impulsivity/Emotional lability	DSM-IV: Hyperactive/Impulsive symptoms	Inattention/Memory problems	DSM-IV: Inattentive
**Velocity in “go”**	0.15	0.2	0.04	0.11
**Acceleration in “go”**	-0.01	0.18	0.01	0.16
**Total distance in “go”**	-0.02	0.07	-0.03	0.16
**Velocity in “stop”**	0.23	0.31*	0.13	0.28
**Acceleration in “stop”**	0.26	0.31*	0.21	*0*.37**
**Total distance in “stop”**	-0.03	-0.06	0.08	0.04

* p < .05

** p < .01

*** p < .001. The scores in italics represent statistically significant correlations after controlling the false discovery rate at q = 0.05 (Benjamini & Hochberg, 1995).

**Table 18:** Values of Spearman’s ρ for correlations between mouse measures and impulsivity/inattention measures at 80% coherency level.

	Impulsivity/Emotional lability	DSM-IV: Hyperactive/Impulsive symptoms	Inattention/Memory problems	DSM-IV: Inattentive
**Velocity in “go”**	0.05	0.22	0.09	0.24
**Acceleration in “go”**	0.11	0.19	0.08	0.22
**Total distance in “go”**	-0.02	0.1	-0.06	0.11
**Velocity in “stop”**	0.1	0.18	-0.01	0.08
**Acceleration in “stop”**	0.14	0.19	-0.04	0.06
**Total distance in “stop”**	-0.16	0.02	-0.05	-0.05

* p < .05, ** p < .01, *** p < .001.

#### Feature subset selection and prediction performance

As in Experiment 1, we applied ridge regression to assess the predictive capacity of keypress and mouse movement measures and indicate the specific features related to the different ADHD subtypes.

#### Feature subset selection

As in the first experiment, we used 10-fold cross-validation approach in both keypress and mouse movement conditions. In the keypress condition, for subscale F (DSM-IV: Hyperactive/Impulsive symptoms), the most important predictors were SD RT in “stop” trials and accuracy in “go” trials. For subscale E (DSM-IV: Inattentive symptoms) most important predictors were accuracy in “go” trials and “stop” trials and mean RT in “stop” trials.

In the mouse movement condition, for subscale F (DSM-IV: Hyperactive/Impulsive symptoms), the most important predictors were mean maximum velocity in “go” and “stop” trials. For subscale E (DSM-IV: Inattentive symptoms) most important predictors were acceleration in “stop” trials and mean RT in “go” trials.

**Fig 6:**
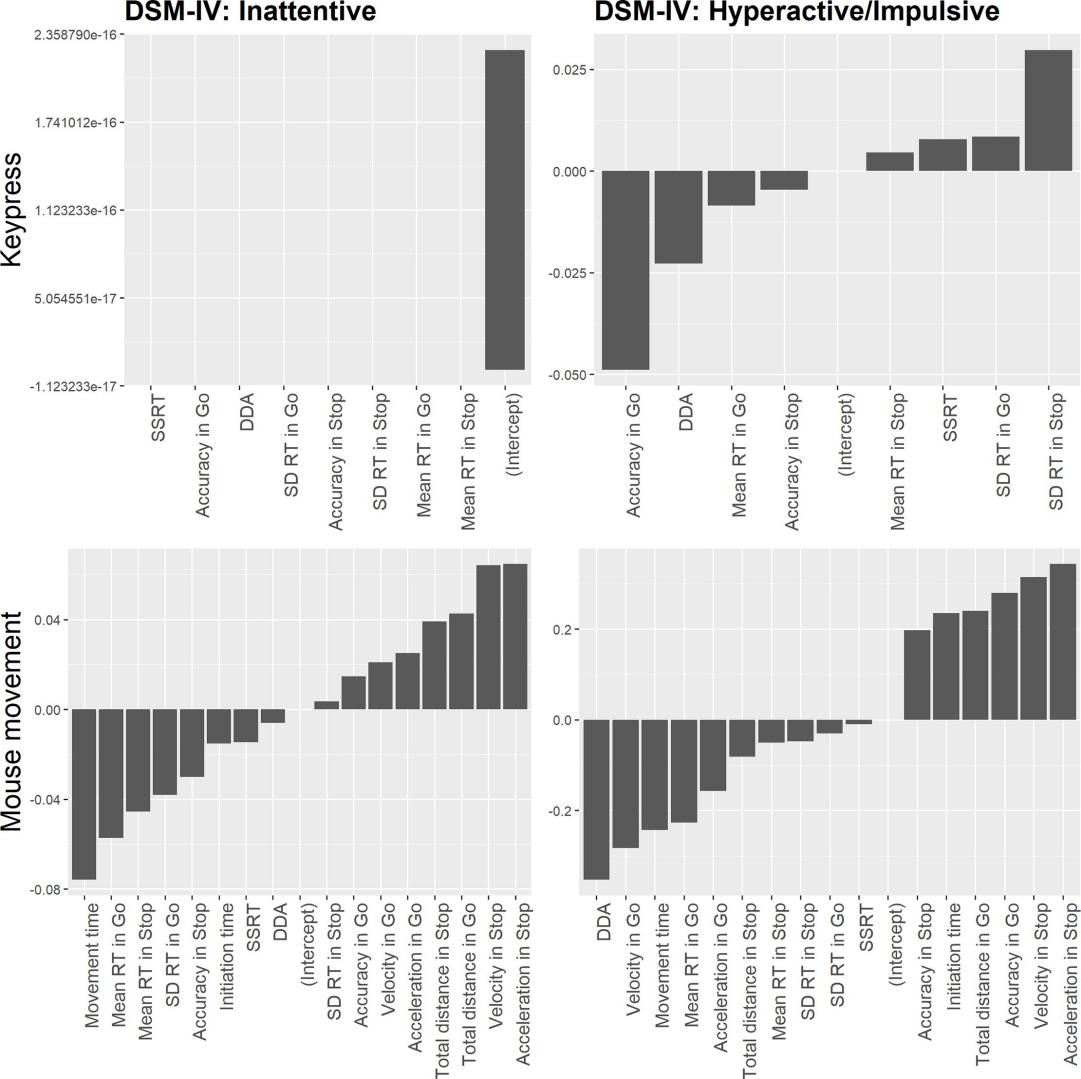
Ridge regression coefficients in keypress and mouse movement conditions. Coefficient value closer to zero indicates less importance. DDA = Direction discrimination accuracy.

A comparison of coefficients’ absolute values between keypress and mouse movement conditions reveals that for both Hyperactive/Impulsive and Inattentive symptoms subscales, features in mouse movement condition had larger absolute values (Fig 6).

#### Evaluation of prediction performance

As in the first experiment, we used 5- and 10-fold nested cross-validation together with bootstrapping. We produced 1000 values of Spearman’s coefficient of correlation between predicted and testing data. Mouse movement-based models were good at predicting the scores on subscales F (DSM-IV: Hyperactive/Impulsive symptoms), A (Inattention/Memory problems) and E (DSM-IV: Inattentive symptoms), while models in keypress conditions were good for predicting scores on the Impulsivity/Emotional lability subscale (Table 19).

**Table 19:** Spearman’s ρ for correlations between predicted and test data in keypress and mouse movement conditions for scores on impulsivity and inattentiveness subscales.

	Impulsivity/Emotional lability	DSM-IV: Hyperactive/Impulsive symptoms	Inattention/Memory problems	DSM-IV: Inattentive symptoms
**Keypress**	0.32 (0.24)	0.21 (0.25)	0.16 (0.27)	-0.12 (0.24)
**Mouse movement**	0.34 (0.22)	0.41 (0.2)	0.4 (0.22)	0.47 (0.16)

Values outside the parentheses are the median values of ρ . Values inside the parentheses are the median absolute deviations of ρ.

#### Discussion

As in Experiment 1, we found very few correlations between RT/accuracy-based measures and impulsivity/inattention subscales. In the keypress condition, we found no significant correlations between performance-based measures and questionnaire subscales describing inclination towards primarily Impulsive or Inattentive subtypes of ADHD. Only the correlation between accuracy in “go” trials and Impulsivity/Emotional lability scores remained significant after false discovery rate control. No SSRT measure had any significant associations with any questionnaire measures in the keypress condition.

In contrast, in the mouse movement condition, strong correlations were found between inclinations towards Impulsive and Inattentive subtypes of ADHD on the one hand and maximum velocity and acceleration in stop trials on the other. These correlations remained significant after controlling for the false discovery rate. Furthermore, ridge regression analysis showed that models using mouse movement measures performed better at predicting scores on the inclination towards different subtypes of ADHD better than models using only RT- and accuracy-based measures. In addition to that, feature selection analysis shows that the absolute values of coefficients in models in mouse movement condition are greater than those in the models for the keypress condition.

These findings suggest that mouse movement measures can supplement SSRT in behavioral impulsivity measures. Interestingly, in the mouse movement condition measures remained significant at a certain level of coherency (50%). This discrepancy, given that the measure in question was calculated based on data from “stop” trials, implies some connection between “stop” and “go” processes. In other words, information pertaining to “go” processes is likely to influence “stop” processes.

Because SSRT is calculated using stop-signal delay (SSD), it is important to identify which cut-off line (i.e., response inhibition criteria) produces the most effective SSRT measures. Unlike the preset method (used in Experiment 1), the staircase method dynamically adjusts stop-signal delay (SSD) as participants make a “correct” or “incorrect” response in a “stop” trial. In Experiment 2, we employed a cut-off line of a 25% of the vertical distance connecting the start position and an end button (Fig 5) to flag an “incorrect” response in a “stop” trial. In this case, the criterion for response inhibition was high in the sense that the cursor moving beyond the 25% cut-off line was treated as a failure of response inhibition.

## General discussion

We compared traditional (keypress) and augmented (mouse movement) versions of the stop-signal task in their associations with questionnaire-based impulsivity/inattention measures. Impulsivity and inattention were assessed through specific scales of Conners’ Adult ADHD Questionnaire.

In Experiment 1, the stop-signal delay was randomly chosen from preset values. We found that keypress measures, including SSRT, had a small association with questionnaire measures, while mouse movement measures had strong and significant correlations. Moreover, mouse movement measures predicted scores on impulsivity subscales better than those in keypress condition. For inclination towards Hyperactive/Impulsive subtype of ADHD, mouse movement measures produced coefficients with greater absolute values than keypress measures, indicating their greater importance for predicting the scores.

In Experiment 2, we employed a staircase method of setting a stop-signal delay, with a more challenging response inhibition criterion (a failure of response inhibition was defined as the cursor crossing 25% of the vertical line between the starting position and the center of response buttons). Mouse movement measures produced strong and significant correlations with scores on the inclination towards Hyperactive/Impulsive subtype of ADHD. SSRT measured in the keypress condition had little to no association with impulsivity measures. Mouse movement measures predicted scores for almost all subscales well, save for Impulsivity/Emotional lability. Similar to Experiment 1, for mouse movement measures, the coefficients’ values were greater.

Taken together, these results suggest that mouse movement measures enhance a stop-signal task in impulsivity and inattention assessment. One of the most popular measures collected in stop-signal tasks, SSRT, was found to have small associations with impulsivity or inattentiveness. Mouse movement measures had, in contrast, strong and significant associations with impulsivity. However, this association was contingent on specific SST design features: it was strong when the inhibition criterion was more challenging (Experiment 2).

The association was particularly strong when the stop-signal delay was devised with the fixed-SSD method (Ma & Yu, 2016). In the context of our experiment, the fixed-SSD method has been shown to be superior to the staircase method when it comes to the assessment of impulsivity (Experiment 1); for inattention, staircase method was better (Experiment 2).

### Theoretical implications

In Experiment 1, we found almost no correlations between mouse movement measures and inattention measures, while in Experiment 2, we found strong correlations between mouse movement measures and inclination towards a primarily Inattentive subtype of ADHD. We speculate that this is related to the specific strategies that a staircase method of setting stop-signal delay prompts a participant to adopt.

As suggested above, the staircase method of SSD setting might be prompting an individual to engage more in suppressing the initiation of responses (proactive inhibitory control) due to its greater reliance on an individual’s performance. Attentional monitoring was found to be a very important part of proactive inhibitory control [75]. Because of that, performance in tasks using the staircase method of setting stop-signal delay might be more representative of inattention, as evidenced by the correlations with inattention measures that appeared in Experiment 2.

If the staircase procedure does indeed prompt an individual to engage in additional proactive response inhibition, the discrepancy between correlations in Experiment 1 (fixed-SSD method) and Experiment 2 can be explained from Toplak and colleagues’ standpoint [24]. According to them, the reason for the discrepancy in correlations between measures collected traditional performance-based tasks and questionnaire-based measurers is that performance-based tasks assess optimal behavior, while questionnaire-based measures assess typical behavior, and are thus more indicative of an individual’s real-world functioning. The staircase procedure, in our case, is more prompting of optimal performance. The fixed-SSD method, on the other hand, is less demanding, allowing an individual to display his typical performance. Because of that, SST using fixed-SSD procedure might be more applicable to gauge individual differences in ADHD-related impulsivity.

### Practical implications

Our findings point to an important potential application of mouse movement measures as a way to indicate different subtypes of ADHD. In Experiment 1, when preset SSDs were used, we found strong and significant correlations between mouse movement measures and inclination towards Impulsive subtype of ADHD (subscale F). Regression models including mouse movement measures have shown significant associations with scores on this subscale as well. On the other hand, keypress measures had significant associations with the inclination towards Inattentive subtype of ADHD (subscale E), indicated by both correlation measures and predictive regression models.

In other words, associations between performance measures and inclination towards different subtypes of ADHD were dependent on which type of performance measures was used (RT/accuracy-based or mouse-movement-specific). Evaluating an individual’s performance with respect to both types of measures can be helpful to indicate his or her inclination towards Impulsive or Inattentive subtype of ADHD.

Another practical advantage of mouse movement tracking as a tool to gauge individual differences in response inhibition lies in a distinct target population. As Lijfijjt and colleagues point out [76], among adults, ADHD is more associated with motor control of inhibition. Even though SSRT is a measure of motor control as well, two important limitations persist: first, SSRT is estimated from response time and accuracy; second, it is an indirect measure. Mouse movement measures do not have these constraints.

Among features of decision making, motor control is of particular interest for ADHD diagnosis, since impairments in it are well known to accompany ADHD [77]. Two areas of executive functions—control of attention and urges, impairment in which is most commonly associated with ADHD, were found to account for almost a half of variance in fine motor skills, and more than a half in gross motor skills among children [78].

These impairments of motor skills include [79], among both children and adults, a heightened muscle tone and lessened ability to inhibit the movement, including legs, hands (i.e. gross motor skills) and thumbs (fine motor skills). In addition to these findings, children with ADHD were found to have more coordination problems, than healthy controls [80]. These problems include manual dexterity as well as aiming and catching (assessed with ball task). Among subtypes of ADHD, motor problems were found to be associated with Inattentive and Combined subtypes (but not Impulsive). Consistent with these findings, our current results highlight the significance of studying motor behavior in adult ADHD.

### Potential and limits of mouse movement augmentation

Because ADHD is a clinical disorder, we recognize the limitation associated with our choice of participants’ demographic. This choice, however, can be justified. Past studies [81] point to the excessive rigidity of the ADHD criteria outlined in diagnostic manuals. This rigidity might potentially exclude individuals with late-onset or subclinical (failing to meet the full clinical definition) ADHD, even though they still experience ADHD-like symptoms and are susceptible to disorders such as cocaine or alcohol abuse [82, 83]. In other words, individuals who are not formally diagnosed with ADHD might still experience ADHD-like symptoms, prompting the need for proper diagnostic tools. Nevertheless, as a future venue for research, we propose contrasting performance in our augmented stop-signal task between ADHD-diagnosed individuals and healthy controls.

In the future, we are also considering studying the associations between performance in the augmented stop-signal task and questionnaire scores for other clinical populations beside ADHD. These populations include but are not limited to, individuals with OCD and substance abuse due to the importance that impulse and urge control play in these disorders [84, 85].

In addition to that, since measures used in this article (velocity, acceleration, and distance) are based on temporal and spatial information about the position of the mouse cursor, the usability of mouse movement measures is inextricable from the quality of timestamp and x-y coordinate registration. For example, if a mouse has a poor sensor, some of the changes in its position can be omitted. Similar concerns can be directed to the surface on which the mouse is placed. Inaccurate reflection of the mouse position and its changes over time can contaminate the mouse movement data and provide an inaccurate picture of an individual’s ability to inhibit his or her responses. These limitations should be considered before using mouse movement in an experimental setting.

### Further extensions

As for any method, mouse movement version of the stop-signal task can be improved in different aspects. One of these aspects is the inclusion of trajectory-related measures of mouse movement. As Maldonado and colleagues have shown [86], trajectory-related measures are more informative of the decision-making process than speed, acceleration, and other similar measures. Future studies can greatly benefit from the inclusion of trajectory-based measures. In our opinion, these measures can help contrast the differences in the decision-making process between healthy and ADHD-prone individuals (or between individuals with different subtypes of ADHD).

### Conclusion

We have found that RT/accuracy-based and mouse movement measures tend to be associated with impulsivity and inattentiveness, respectively. Our findings also point to the influence of stop-signal delay setting method on associations with impulsivity and inattention.

Our findings highlight the need for expanding the stop-signal task measures of ADHD-related impulsive traits in under-researched populations, such as college students, to include mouse movement measures as a part of SST’s measures.

We also consider research into the differences between behaviors in stop-signal tasks using preset SSDs and staircase method of SSD to be very important. The discrepancy in associations between mouse movement measures and impulsivity measures in Experiments 1 (which uses preset SSDs) and 2 (which uses staircase method) point to the importance of SSD setting method for associations between behavioral and questionnaire-based measures of impulsivity. While we provide a potential explanation of this discrepancy above, further investigation is needed.

It should be noted, however, that traditional stop-signal task’s measure–SSRT and other response time/accuracy-based measures—remain useful in regards to inhibition. In the diagnostic process, mouse movement measures should be used together with–not instead of–stop-signal reaction time.

In conclusion, this study has yielded important findings in the potential of mouse movement measures for detection of individual differences across the ADHD spectrum. It has also highlighted new venues for research into the influence of SST’s parameters, such as method of stop-signal delay setting or different response inhibition criteria, on an individual’s stopping behavior and associations between performance and questionnaire-based measures. We suggest that future research on ADHD and inhibition will benefit from incorporating mouse movement measures.

## Supporting information

S1 Text Supplementary methods. (DOCX)

S1 Fig Comparison of scores on different CAARS subscales between two conditions in Experiment 1. (TIF)

S2 Fig Comparison of scores on different BDEFS sections between two conditions in Experiment 1. (TIF)

S3 Fig Comparison of scores on different CAARS subscales between two conditions in Experiment 2. (TIF)

S1 Data (RAR)
