# Supplementary material for: Mouse movement measures enhance the stop-signal task in adult ADHD assessment
Source: PLoS One. 2019 Nov 26;14(11):e0225437. doi: 10.1371/journal.pone.0225437 (PMC6880625; doi:10.1371/journal.pone.0225437)
Supplement: S1 Text — (DOCX) [file pone.0225437.s001.docx]

**Supplementary methods**

## **Connors Adult ADHD Questionnaire (CAARS)**

After completing the Stop-signal task, participants were required to complete CAARS [52]. The Conners Adult ADHD questionnaire – self-report long version (CAARS-S: L) is a widely accepted ADHD assessment tool. CAARS is a 66-item measure that asks participants to indicate how accurately are the statements from the questionnaire describing of participants’ personal feelings from the past two weeks till present time [52]. Responses are coded on a scale of 0 to 3. Higher scores represent a statement’s stronger indication of a participants’ current condition. Internal consistency estimates for CAARS range from .79 to .90 for all subscales in the total sample. Validity estimates fall in a range from .82 to .93 for all subscales in the total sample.

Scores on CAARS are mapped onto 8 subscales. Higher scores on subscale A (Inattention/Memory problems) are associated with concentration, planning, completing tasks, forgetfulness troubles, as well as absent-mindedness and disorganization above the norm. Subscale B (Hyperactivity/Restlessness) designates problems with fidgeting, staying on the same task for long periods of time, and elevated overall restlessness. Subscale C (Impulsivity/Emotional lability) signals about low frustration tolerance, mood changes, being easily angered or irritated, and an overall inclination to engage in more impulsive acts than others. Higher scores on subscale D (Problems with self-concept) are representative of lower self-esteem and self-confidence, as well as poor social relationships. DSM-IV subscales, namely, E (DSM-IV: Inattentive symptoms), F (DSM-IV: Hyperactive-Impulsive Symptoms), G (DSM-IV: ADHD symptoms total), reflect behavior found among Inattentive, Hyperactive-Impulsive and Combined subtypes of ADHD respectively. Finally, subscale H (ADHD index) is considered to be a general indication of ADHD symptoms severity necessary for clinical diagnosis. Individual scores obtained for these subscales served as dependent variables.

## **Barkley Deficits in Executive Functioning Scale (BDEFS for Adults)**

In Experiment 1 participants were also required to complete BDEFS questionnaire. BDEFS is a well-established measure of executive function. BDEFS is an 89-item questionnaire with a four-point response scale: Never/Rarely, Sometimes, Often, or Very Often. These responses indicate how often participants experience certain symptoms. BDEFS questions are mapped onto five subdomains: Self-Management to Time, Self-Organization/Problem Solving, Self-Restraint/Inhibition, Self-Motivation, and Self-Regulation of Emotions.

Higher scores on subdomain “Self-Management to Time” describe procrastination, difficulty using foresight to plan events, difficulty setting goals, and difficulties with self-discipline. Internal consistency coefficient (Сronbach’s α) for this subdomain equals .949[53]. Subdomain Self-Organization/Problem Solving describes inclination to being easily confused, inability to sustain concentration, difficulty in thought expression and thought organization (Сronbach’s α = .958). Higher scores on subdomain Self-Restraint/Inhibition (Сronbach’s α = .93) indicate low tolerance for frustration, troubles inhibiting emotions and comments to other or changing behavior after being given feedback about mistakes. This subdomain is also indicative of an inability to understand others’ perspectives about a problem or situation. Self-Motivation (Сronbach’s α = .914) describes troubles in reward delaying as well as resisting urges to engage in leisurely activities and being dependent on others as well as troubles in contributing effort at the same rate as others. Finally, Self-Regulation of Emotion (Сronbach’s α = .946) describes pessimistic attitudes, overreacting emotionally and troubles experiencing positive emotions. Overall internal consistency coefficient for BDEFS is equal to .918.

**Legends to Supplementary Figures**

**Supplementary Figure 1**

**Comparison of scores on different CAARS subscales between two conditions in Experiment 1.**

Distributions are smoothed with local regression (LOESS) algorithm, a non-parametric method which fits simple least squares regression models to the localized subsets of data [63]. P-values indicate the results of Kolmogorov-Smirnov test.

**Supplementary Figure 2**

**Comparison of scores on different BDEFS sections between two conditions in Experiment 1.**

Distributions are smoothed with local regression (LOESS) algorithm. P-values indicate the results of Kolmogorov-Smirnov test.

**Supplementary Figure 3**

**Comparison of scores on different CAARS subscales between two conditions in Experiment 2.**

Distributions are smoothed with local regression (LOESS) algorithm. P-values indicate the results of Kolmogorov-Smirnov test.
