# Supplementary figures and images for: Mouse movement measures enhance the stop-signal task in adult ADHD assessment
Source: PLoS One. 2019 Nov 26;14(11):e0225437. doi: 10.1371/journal.pone.0225437 (PMC6880625; doi:10.1371/journal.pone.0225437)

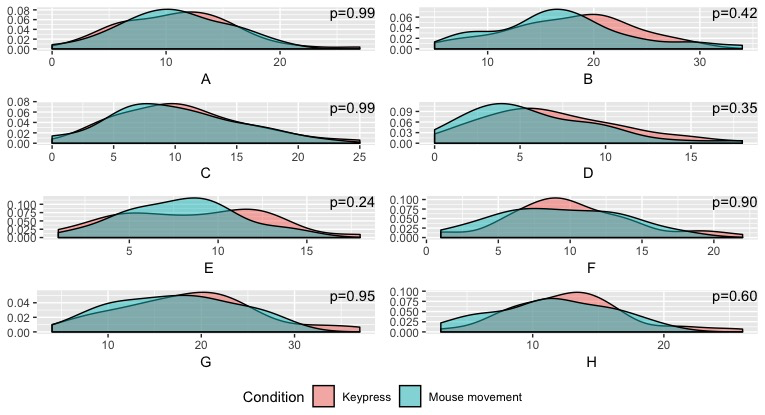

Supplement: S1 Fig — (TIF) [file pone.0225437.s002.tif]

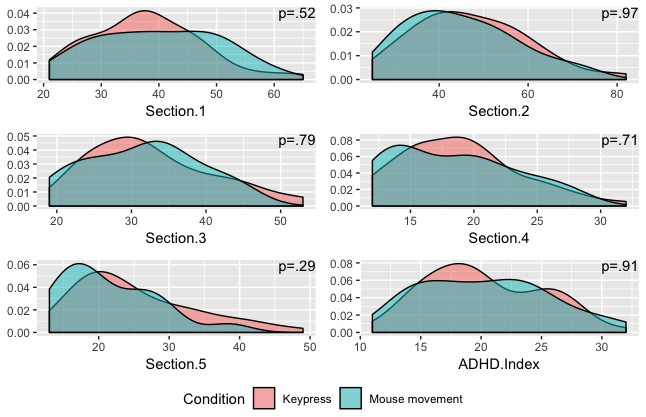

Supplement: S2 Fig — (TIF) [file pone.0225437.s003.tif]

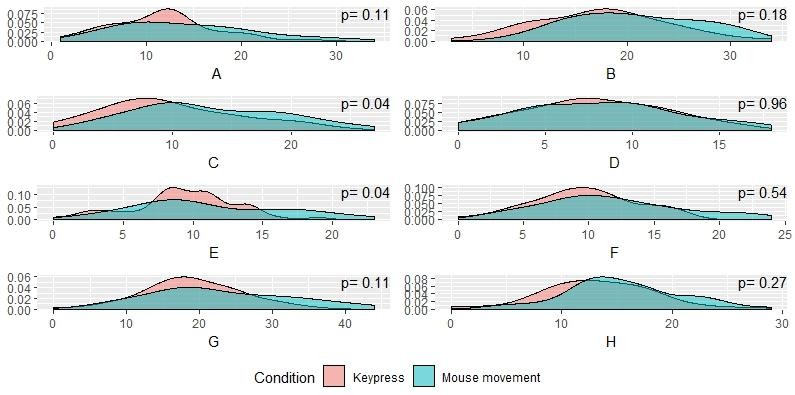

Supplement: S3 Fig — (TIF) [file pone.0225437.s004.tif]
